# Chemical compositions, antioxidant, antimicrobial, and mosquito larvicidal activity of *Ocimum americanum L.* and *Ocimum basilicum* L. leaf essential oils

**DOI:** 10.1186/s12906-023-04214-2

**Published:** 2023-10-28

**Authors:** Ganesan Mahendran, Sornkanok Vimolmangkang

**Affiliations:** 1 Department of Pharmacognosy and Pharmaceutical Botany, Faculty of Pharmaceutical Sciences, Chulalongkorn University, Bangkok, 10330 Thailand; 2 Center of Excellence in Plant-Produced Pharmaceuticals, Chulalongkorn University, Bangkok, 10330 Thailand

**Keywords:** *Ocimum*, Basil leaf, Antibacterial, Flavor, Aromatherapy

## Abstract

**Background:**

*Ocimum americanum L. (O. americanum)* and *Ocimum basilicum* L. (*O. basilicum*) are highly valued aromatic medicinal plants. Their leaves are widely used as spices in traditional cuisine. Their essential oils (EOs) are extensively used in food, cosmetic, and pharmaceutical industries. This study aimed to investigate the main chemical profiles of *O. americanum* and *O. basilicum* leaf EOs and assess their effects on antibacterial, antioxidant, and larvicidal properties.

**Methods:**

EOs were extracted from the leaves of *O. basilicum* and *O. americanum* using steam distillation in a Clevenger-type apparatus. The chemical constituents of the EOs were analyzed using gas chromatography–mass spectrometry. 2,2-Diphenyl-1-picrylhydrazyl (DPPH), ferric reducing antioxidant power (FRAP), and metal-chelating techniques were used to assess the free-radical scavenging capability of the oils. The extracted oils were also tested for their antibacterial activities via a disk-diffusion test and the broth microdilution method. Furthermore, the mosquito larvicidal (*Aedes aegypti*) activity was tested using standard protocols.

**Results:**

Camphor (33.869%), limonene (7.215%), longifolene (6.727%), caryophyllene (5.500%), and isoledene (5.472%) were the major compounds in *O. americanum* leaf EO. The EO yield was 0.4%, and citral (19.557%), estragole (18.582%) camphor (9.224%) and caryophyllene (3.009%) were the major compounds found among the 37 chemical constituents identified in *O. basilicum* oil. *O. basilicum* exhibited a more potent antioxidant activity in DPPH, FRAP, and 2,2′-azino-bis(3-ethylbenzothiazoline-6-sulfonic acid tests than *O. americanum*. The zones of inhibition and minimum inhibitory concentration of the oils in the microdilution and disk diffusion methods were 8.00 ± 0.19 mm to 26.43 ± 2.19 mm and 3.12–100 µg/mL, respectively. At 400 ppm, *O. basilicum* and *O. americanum* EOs demonstrated larvicidal activity, with mortality ratios of 73.60% ± 0.89% and 78.00% ± 1.00%, respectively. Furthermore, after 30 min of exposure to *O. americanum* and *O. basilicum EOs*, the larval death rates were 73.60% ± 0.89% and 78.00% ± 1.00%*,* respectively.

**Conclusions:**

The findings revealed that the EOs extracted from the leaves of *O. basilicum* and *O. americanum* exhibited reasonable antioxidant, antibacterial, and mosquito larvicidal potentials, and can be used as alternative medicine for the treatment of human health and larvicidal mosquito control.

## Background

Plants are essential for maintaining the ecological dynamics of ecosystems. They also offer numerous substances with potential healing properties [1, 2]. Medicinal plant extracts and essential oils (EOs) are factories of valuable natural bioactive compounds, which are widely used in the food, pharmaceutical, nutraceutical, and cosmetic industries for their fragrance, taste, and therapeutic capabilities [3–6]. Terpenes and their oxygenated derivatives, benzoids, and phenylpropanoids are volatile, complex mixtures of EOs [4, 7]. In general, EOs are often mixtures of large amounts of two or three compounds as the main components (> 20%–95%) these compounds and others that may be present in very small amounts.

Free radicals, such as reactive oxygen species (ROS), form in redox processes, and are the leading cause of degenerative diseases, such as stroke, rheumatoid arthritis, diabetes, inflammation, aging, cancer, and neurological disorders of the human body [2, 8]. ROS have been associated with lipid oxidation, peroxidation, and DNA mutations of proteins [9]. Antioxidants include any substance that can remove ROS. Given their resonance-stabilizing effect, phenolic compounds can effectively scavenge free radicals [10]. Interest in the search for new plant-based herbal medicines with antioxidant properties has grown in modern times.

Bacterial infections are increasing nowadays. As with the use of antibiotics for bacterial treatment, the rate of therapeutic drug failure is increasing due to the development of resistance of microbial strains. Despite the medical advances in treatment of infectious diseases, chemotherapy and immunization remain serious health problems [11]. The resistance of microorganisms to antimicrobial drugs necessitates the research on new medications with a potential antimicrobial activity [12]. Studies have focused on plant-derived EOs, which contain various volatile bioactive constituents that can act as therapeutics in humans.

*Aedes aegypti* L. is the vector of arboviruses that cause diseases, such as yellow fever, dengue fever, chikungunya, and zika in tropical regions [13–15]. Rapid urbanization, the lack of infrastructure, and poor sanitation favor the spread of female *Aedes aegypti* mosquito, which is resistant to pesticides and commercially available repellents [13, 15]. In addition, the disadvantages of chemical pesticides, such as pollutants, and harm to nontarget species, have limited their widespread use [16]. Therefore, alternative pesticides with high efficacy, minimal environmental impact, and low toxicity to humans must be developed to control mosquitoes. Currently, mosquito control programs focus on the eradication of mosquito larvae with herbal remedies [17]. In this context, plant-derived EOs have attracted considerable attention as potential sources of mosquito repellents and larvicides [18].

The genus *Ocimum* (basil) comprises 160 species and is the major species in Lamiaceae globally. *Ocimum* is called “the king of herbs” because of its enormous applications in traditional medicine, pharmaceuticals, and cosmetic industry [19, 20]. Several species of *Ocimum,* such as *O. basilicum*, *O. gratissimum*, *O. tenuiflorum*, *O. americanum*, *O. kilimandscharicum,* and *O. micranthum,* are cultivated for their high-value fragrance, flavor, and medicinal properties [21]. *Ocimum* has been traditionally used to treat a variety of ailments, including rheumatism, epilepsy, paralysis, diarrhea, sunstroke, influenza, high fever, gonorrhea, abdominal pains, mental illness, colds, and coughs; it also possesses antipyretic, antiemetic, stomatic, antihelmintic, and antimalarial activities [22]. Furthermore, *Ocimum* leaves have been investigated as a food and flavoring agent because of their aromatic properties [23]. The EOs and aromatic fragrance of *Ocimum* leaves have therapeutic potentials, especially antimicrobial, antioxidant, insecticidal, and nematocidal properties [24]. Interestingly, *Ocimum* EOs are rich in terpenoids and phenylpropanoids, such as methyl cinnamate, linalool, thymol, camphor, citrol, eugenol, and geraniol, which are important active constituents that are variable and influenced by environmental factors [23–25].

*Ocimum basilicum* L. is an aromatic herb and main commercial crop with numerous biological uses [26]. This plant is used as a supplement in the perfume, pharmaceutical, food, aromatherapy, and cosmetic industries [27]. EOs and aromatic leaves have been used in plant-based healthcare since ancient times in traditional systems of medicine [28]. *Ocimum basilicum* has an aromatic fragrance distinguished by its chemotypes, especially methyl chavicol-, linalool-, eugenol-, methyl eugenol-, and methyl cinnamate-rich chemotypes, which have been documented in India [23]. Earlier studies have reported that the EOs extracted from *O. basilicum* exhibited potential antimicrobial, fungicidal, antioxidant, antiviral, antiproliferative, anti-inflammatory, antispasmodic, and sedative properties [29].

*Ocimum americanum* L. (syn. *O. canum* Sims.) is another renowned species of *Ocimum*, and it is commonly spread through India and tropical Africa. It is also known as “hairy basil.” Preparations achieved from its aerial parts are repetitively used in folk remedies for the treatment of insomnia and anxiety [30]. In Nigeria, *O. americanum* leaf decoction or infusion is used to control fever, coughs, colds, piles, and diabetes [31]. In Yoruba tribals, *O. americanum* is used to prepare soup given its aroma and flavor [32]. *O. americanum* acetone extract inhibited neurotoxins that caused brain damage in rats [33]. With these regards, the current work was conducted to determine the chemical profiles of EOs extracted from *O. basilicum* and *O. americanum* leaves and investigate their antibacterial, antioxidant, and ant mosquito larvicidal properties.

## Methods

### Plant material and extraction of EOs

Fresh leaves of *O. basilicum* and *O. americanum* were collected from an agricultural field (permission was obtained from the landowner) in Coimbatore, Tamil Nadu, India. The identifications of the leaves were confirmed by Dr. Jagathes Kumar, Assistant Professor, and Botanist, Department of Botany, Sri Vijay Vidyalaya College of Arts & Science. The voucher specimens (LO005 and LO006) were deposited at the herbarium center. The collected leaves of *O. basilicum* and *O. americanum* were shade dried at room temperature. The EOs were then isolated from 500 g dried leaves through hydrodistillation for 3 h using a Clevenger apparatus. The extracted oils were dried with anhydrous sodium sulfate (1.0 g), separated, and stored at 4 °C for further use.

### Chemical constituents of EOs

After the separation of EOs, their chemical composition was determined by gas chromatography–mass spectrometry (GC–MS) using a Clarus 680 GC–MS (PerkinElmer®) equipped with a capillary column (30 m × 0.25 mm) of diphenyl dimethyl polysiloxane model Elite-5 MS. The oven temperature was slowly increased from 60 C to 300 C at the rate of3 °C min^–1^. The temperatures of injection and mass were set at 220 C and 240 C, respectively. The EOs (1.0 µL) were injected and with a split ratio of 1:200. The flow rate of helium gas was 1 mL/min. GC–MS was performed using the same chromatography and a mass spectrometer. The GC conditions were also the same. The chemical constituents were identified using the retention time and mass spectra in the Wiley and NIST libraries of GC–MS and literature.

### Antioxidant activities of EOs

#### 2,2-Diphenyl-1-picrylhydrazyl (DPPH) radical scavenging activity

The DPPH free-radical scavenging potential of EOs was measured as described by Blois [34] but with minor changes. A total of 300 µL EO of varying concentrations (20–100 µg/mL) was mixed with the DPPH solution (3 mL 0.1 mM). The oil mixtures were kept in a darkroom for 30 min, and the color reduction by the EOs of the stable DPPH radical was measured at 517 nm on a spectrophotometer. Ascorbic acid, rutin, and butylated hydroxytoluene (BHT) were used as standards. DPPH was calculated using the equation below:

$$\%\, \mathrm{inhibition}=\frac{\mathrm{Control\, OD }-\mathrm{Sample \,OD}}{\mathrm{Control\, OD}} \times 100$$

The concentration of oil that reduced the DPPH solution by 50% (half-maximal inhibitory concentration (IC_50_)) was calculated.

#### Measurement of 2,2′-azino-bis (3-ethylbenzothiazoline-6-sulfonic acid (ABTS^•^) radical cation assay

The ABTS^*•*^ radical cation decolorization potential of EO from *O. americanum* and *O. basilicum* was measured in accordance with the work of Re et al. [35]. ABTS radical was produced by ABTS (7 mmol/L) with potassium persulfate (2.4 mM) in the dark for 12–16 h. Then, the ABTS solution was diluted in ethanol (1:89 v/v) to give an absorbance of 0.700 ± 0.02 at 734 nm. Triplicates of 10 μL essential oil (20 mg/mL in DMSO) and Trolox (concentration 0–15 μM) were added to 1 mL of ABTS solution. The reaction mixture was incubated at 30 °C for 30 min and the absorbance was measured at 734 nm. The total antioxidant activity was validated as Trolox equivalent µM/g oil. Ascorbic acid was used as a standard.

### Ferric reducing antioxidant power (FRAP) assay

The FRAP-reducing antioxidant power potentials of *O. basilicum* and *O. americanum * EOs were esteemed following the work of Pulido et al. [36]. 900 µL of FRAP reagent was mixed with 90 µL of distilled water and 50 µL essential oil (20 mg/mL). The essential oil and blank were incubated at 37 °C for 30 min. After incubation, the optical density was taken at 593 nm using a spectrophotometer. Methanolic solutions with known Fe (II) (FeSO_4_·7H_2_O) concentrations, ranging from 100 to 2000 µmol/L were used for the preparation of the calibration curve. FRAP was indicated as mM Fe (II) equivalent/g oil. Ascorbic acid was used as a standard.

### Metal-chelating activity

The chelation of ferrous ions by *O. basilicum* and *O. americanum* EOs was assessed in accordance with the method of Dinis et al. [37]. 100 µL of essential oil (20 mg/mL), 600 µL distilled water, and 100 µL ferrous chloride (2 mmol/L) were mixed, shaken vigorously, and incubated for 30 s. Then, 200 µL ferrozine (5 mmol/L) was added to the above mixture and incubated for 10 min at room temperature and the absorbance was recorded at 562 nm with a UV–vis spectrophotometer. EDTA (0–2 µg) was used as a standard for the preparation of the calibration curve. The metal chelating ability of antioxidants was expressed as mg Ethylenediaminetetraacetic acid (EDTA) equivalent/g oil.

### Antibacterial activity

The antibacterial activity of *O. basilicum* and *O. americanum* EOs was assessed by two tests: (i) the c and (ii) broth microdilution (determination of minimum inhibitory concentration (MIC) and minimum bactericidal concentration (MBC)) tests. The antibacterial efficacies of *O. basilicum* and *O. americanum* oils were tested against gram-positive bacteria, namely, *Bacillus subtilis* (MTCC-441), *Streptococcus pyogenes* (MTCC 1928), *Enterococcus faecalis* (MTCC 439), and *Staphylococcus aureus* (MTCC-96), and gram-negative bacteria, including *Escherichia coli* (MTCC-724), *Klebsiella pneumoniae* (MTCC-432), *Proteus vulgaris* (MTCC-426), *Salmonella paratyphi* (MTCC-735), *Aeromonas hydrophila* (MTCC-7646), *Pseudomonas aeruginosa* (MTCC 2453), and *Enterobacter aerogenes* (MTCC 39).

### Diameter of the zone of inhibition (ZI) by disk diffusion

The antibacterial activities of *O. basilicum* and *O. americanum* EOs were evaluated using the paper-disk diffusion method, in accordance with the work of Mahendran and Ranjitha Kumari [38]. Muller–Hinton (Himedia, India) agar plates were prepared and stored at 20 °C ± 2 °C. A test bacterial suspension (100 µL) containing 10^8^ CFU/mL of each bacterial suspension was dispensed and uniformly spread above the sterile plates using a glass spreader. Then, 20 µL (5, 10, and 20 mg/mL dissolved in DMSO) of each EO was loaded onto a sterile paper disk (6 mm). Oil-loaded paper disks were placed on the surface of the inoculated plates, and the plates were incubated at 37 °C ± 2 °C for 24 h. Streptomycin (20 µg/disk) and cefotaxime (20 µg/disk) disks were used as a reference (positive control) and DMSO (negative control). The experiment was carried out on three plates (in triplicate) for each organism. The ZI diameter was measured in millimeters (mm). The results were presented as mean ± standard deviation (SD).

### Broth microdilution method for MIC and MBC

The MIC and MBC of *O. basilicum* and *O. americanum oils* were evaluated using the microdilution method, in accordance with the work of Duarte et al. [39]. Briefly, 100 µL twofold serially diluted (100 µg/mL to 0.781 µg/mL) oils of *O. basilicum* and *O. americanum* were deposed in each well in a 96-well plate. Then, 100 µL bacteria at 10^6^ CFU were added to all wells. After incubation at 37 °C overnight, 40 µL (200 µg/mL) *p*-iodonitrotetrazolium violet (INT) was deposed to all wells for the evaluation of bacterial growth. Bacterial growth was shown by detecting the reduction of yellow to red formazan after 2 h of incubation at 37 °C [40]. To analyze the MBC, we sub-cultured 10 µL of each bacterial culture on Muller–Hinton plates for 24 h.

### Mosquito larvicidal bioassay

The eggs of *Aedes aegypti* were acquired from the Center for Research in Medical Entomology, Madurai, India. The larvae were fed with yeast powder and dog biscuits at a 1:3 ratio. A total of 10% sucrose with chicks for blood meal was nourished for adults. Mosquitoes were maintained at 70%–85% relative humidity and 28 °C ± 2 °C temperatures with 12 h light.

The larval mortality was measured in accordance with the work of Panneer Selvam et al. [41] with slight modifications. A total of 25 larvae at the 3^rd^ and 4^th^ instar stages were separately kept in 200 mL paper cups containing 99 mL water added with 1 mL of essential oil at various concentrations (25, 50, 100, 200, and 400 ppm) dissolved in 1 mL of DMSO. The mortality was detected after 24 and 48 h. Toxicity (mortality) and larvicidal activities were described using 50% lethal concentration (LC_50_) and LC_90_ at 50% concentration of essential oil showed mortality. 95% confidence limit levels were calculated by probit analysis (SPSS ver. 26).

### Statistical analysis

All the biological activities of both EOs were repeated in triplicate, and the results were given as mean ± SD and calculated using SPSS version 26. The IC_50_ value was investigated statistically with a one-way analysis of variance followed by Duncan’s multiple range test. Probit analysis was used to estimate the LC_50_ and LC_90_ lethal concentrations with a 95% confidence limit (CL) were all calculated.

## Results

### Chemical constituents of *O. basilicum* and *O. americanum* EOs

**Fig. 1:**
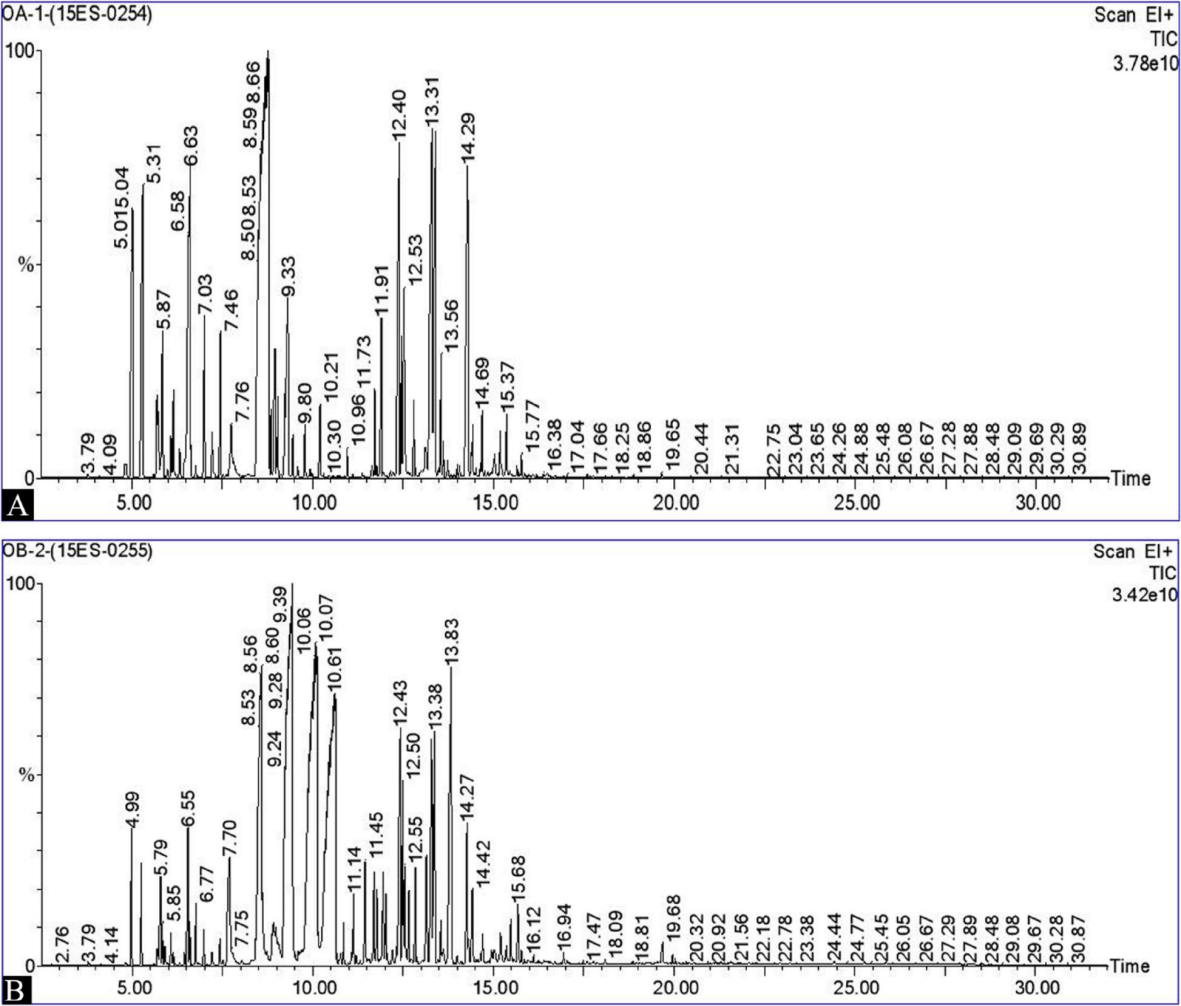
GC–MS chromatogram of leaf EOs from *Ocimum* species. **A** *O. americanum* **B*** O. basilicum*. The EOs obtained from *O. americanum* had 34 compounds and contained camphor (33.86%), limonene (7.21%), longifolene (6.72%), veridiflorol (5.85%), isoledene (5.472), and α-pinene (5.19%) as the main chemical components (Table 2 and Fig. 2)

Hydrodistillation of *O. basilicum* and *O. americanum* yielded EOs about 0.6% (3.2 mL) and 0.4% (2.3 mL), respectively. The EOs of *O. basilicum* and *O. americanum* were investigated, and their chromatographic profiles are shown in Fig. 1A and B, respectively. Their chemical compositions from the experiment were compared with those of a previous study (Tables 1 and 2). The results exhibited that 37 and 34 compounds were identified, and they accounted for 81.141% and 100% of the oil, respectively, in *O. basilicum* and *O. americanum.* As shown in Table 1, the main components of *O. basilicum* were 3,7-dimethyl-,(Z)-(-citral) (19.557%), estragole (18.582%), camphor (9.224%) and caryophyllene (3.009%).

**Table 1:** Chemical composition of *O. basilicum* oil

**Peak**	**Retention time (min)**	**Mass**	**Compounds**	**Area (%)**	**[Ref]**^a^
1	4.994	136	α-Pinene	1.010	1, 3
2	5.264	136	Camphene	0.729	1, 3
3	5.795	128	1-Octen-3-ol	0.771	4
4	5.850	136	β-Pinene	0.246	1, 3
5	6.550	136	D-Limonene	1.287	2
6	6.600	154	Eucalyptol	0.251	1, 2, 3
7	6.770	136	α-Ocimene	0.391	
8	6.990	136	3-Carene	0.235	7
9	7.700	154	*Cis*-β-Terpineol	2.715	
10	8.596	152	Camphor	**9.224**	1, 2, 3
11	8.921	152	(-)-Myrtenol	0.781	1, 3
12	8.981	152	( +)- trans-Limonene oxide	0.757	7
13	9.441	148	Estragole	**18.582**	5
14	10.071	152	2,6-Octadienal, 3,7-Dimethyl-, (Z)- (β – citral)	**19.557**	5
15	10.862	204	α- farnesene	0.250	1, 3
16	11.137	166	4,8-dimethyl-nona-3,8-dien-2-one	0.479	
17	11.452	154	(. ± .)-Lavandulol,	0.855	4
18	11.707	180	Geranyl vinyl ether	0.734	
19	11.952	204	Cadrene	0.766	
20	12.427	204	Caryophyllene	**3.009**	1
21	12.497	204	*trans*- α-Bergamotene	1.470	2
22	12.547	204	*iso*ledene	0.685	
23	12.662	204	α-Cubebene	0.560	1, 3
24	12.847	204	α-Caryophyllene	0.793	3
25	13.153	204	Aromadendrene	1.311	6
26	13.298	204	(-)-Iso aromadendrene -(V)	2.268	
27	13.378	204	Patchoulene	2.300	
28	13.553	204	Longifolene	0.309	
29	13.623	204	(-)-α-panasinsen	0.199	
30	13.833	222	Levomenol	**4.888**	
31	14.273	222	(-)-Globulol	1.282	
32	14.418	220	Caryophyllene oxide	0.219	2
33	14.708	166	Cyclohexane, Butylidene	0.761	
34	15.193	222	Epiglobulol	0.443	
35	15.484	222	α -Bisabolol	0.320	2
36	15.684	222	Veridiflorol	0.495	2
37	19.680	222	Ledol	0.209	
			**Total**	**81.141**%	

^a^1 (Abou El-Soud, Deabes, Abou El-Kassem, & Khalil 2015)

2 (Hussain, Anwar, Hussain Sherazi, & Przybylski 2008)

3 (Ismail 2006)

4 (Politeo, Jukic, & Milos 2007)

5 (Beatovic et al. 2015)

6 (Avetisyan et al. 2017), and

7 (Chalchat & Özcan 2008)

**Table 2:** Chemical composition of *O. americanum* oil

**Peak**	**Retention time (min)**	**Mass**	**Compounds**	**Area (%)**	**[Ref]**^a^
1	5.039	136	α-Pinene	**5.195**	1, 2, 3
2	5.315	136	Camphene	4.674	2
3	5.710	204	α -farnesene	0.972	
4	5.755	128	1-Octen-3-ol	0.398	5
5	5.870	136	β-Pinene	1.694	1,2,3
6	6.165	136	α-Phellandrene	0.773	
7	6.625	136	Limonene	**7.215**	1,3
8	7.025	136	3-Carene	1.673	
9	7.235	154	*Cis*-β-Terpineol	0.535	3
10	7.460	136	Cyclohexene	1.433	
11	7.756	154	Eucalyptol	1.220	1,2,3
12	8.771	152	Camphor	**33.869**	1,2,3
13	8.856	154	Iso Borneol	0.388	2
14	8.971	154	Borneol	1.394	1
15	9.046	154	α-terpinenol	0.671	1,3
16	9.241	148	Estragole	0.937	5
17	9.326	152	(-)-Myrtenol	3.206	1
18	9.796	152	Octadienal	0.476	
19	10.212	204	Naphthalene	0.691	2
20	11.727	204	Copaene	0.696	1
21	11.907	204	Cadrene	1.508	
22	12.402	204	Caryophyllene	**5.500**	
23	12.462	204	β-Caryophyllene	1.060	4
24	12.532	204	Aromadendrene	1.696	3
25	12.803	204	α-Caryophyllene	0.661	5
26	13.118	204	α-Cubebene	0.450	
27	13.308	204	Longifolene	**6.727**	
28	13.403	204	Isoledene	**5.472**	
29	13.558	204	Epizonarene	0.992	
30	14.293	222	Veridiflorol	**5.858**	
31	14.693	222	Humulane	0.541	5
32	15.038	222	α-cadinol	0.341	1
33	15.189	222	Ledol	0.512	
34	15.369	222	γ-Muurolene	0.572	
			**Total**	**100%**	

^a^1 (Singh, Tewari, Pande, & Singh 2013)

2 (Bhatt, Tewari, Pande, & Rana 2018)

3 (Matasyoh, Bendera, Ogendo, Omollo, & Deng 2006)

4 (Coulibaly, Hema, Sawadogo, Toe, Kiendrebeogo, & Nébié 2023), and

5 (Wungsintaweekul, Sitthithaworn, Putalun, Pfeifhoffer, & Brantner 2010)

**Fig. 2:**
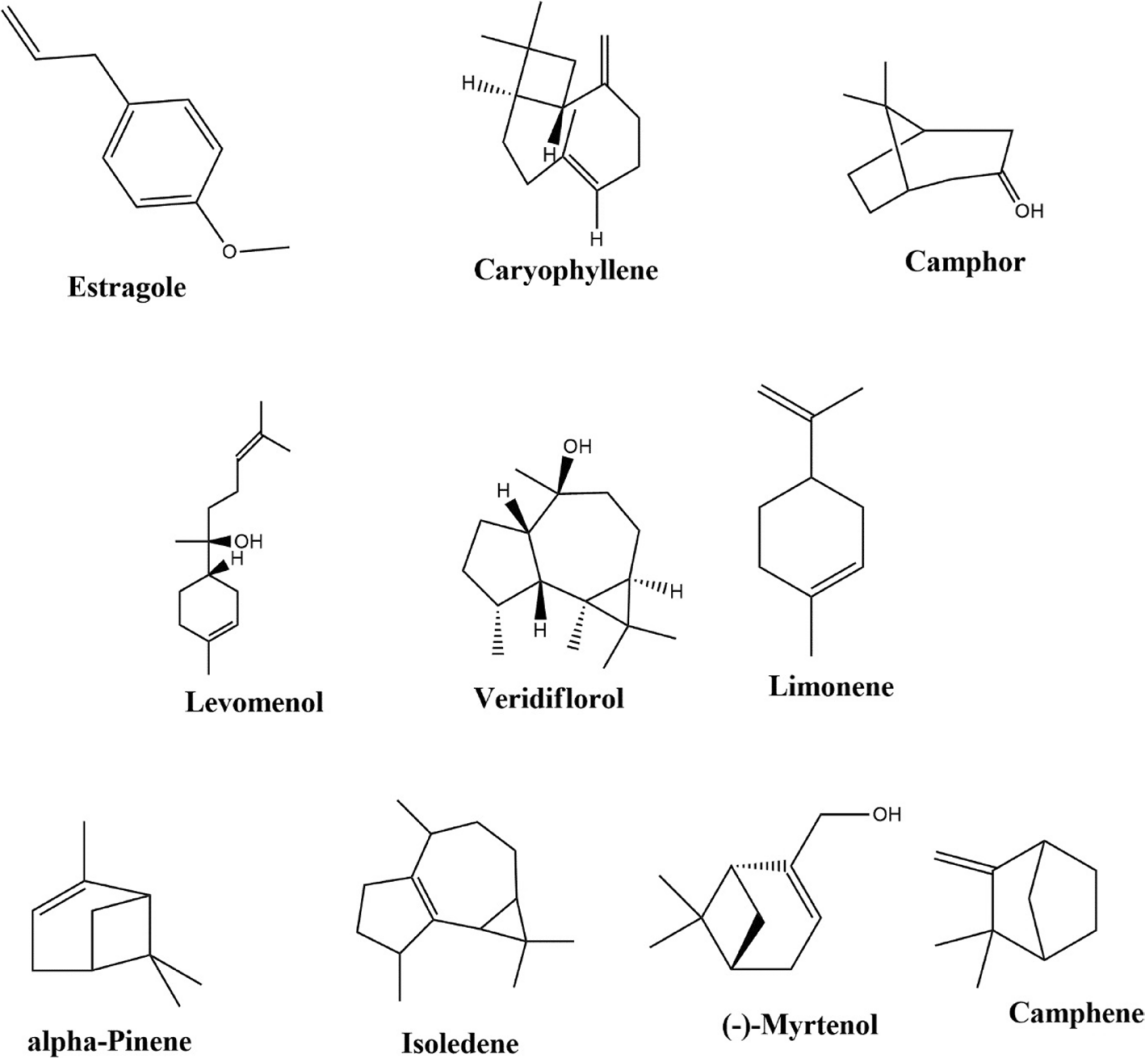
Predominant phytochemicals are present in the EOs of *O. americanum* and *O. basilicum* leaves

### Antioxidant activity

In the present research, the antioxidant activities of *O. basilicum* and *O. americanum* EOs were determined via four different investigations, including DPPH, FRAP, ABTS, and metal-chelating assays.

The present results revealed that *O. basilicum* oil had more noticeable activity than *O. americanum* oil because its IC_50_ value was lower (11.56 ± 0.89 µg/mL) than that of *O. americanum* (13.42 ± 1.03 µg/mL). However, the standards, which included ascorbic acid (3.72 ± 0.60 µg/mL), rutin (4.65 ± 0.52 µg/mL), and BHT (7.89 ± 0.49 µg/mL), exhibited higher activities. 

The Trolox equivalent antioxidant capacity (TEAC) was also assessed by neutralizing the radical cation of ABTS. The ABTS activity of *O. basilicum* EO (2842.12 ± 10.39 µM TEAC/g oil) showed more potency than that of *O. americanum* (2085.07 ± 7.43 µM TEAC/g oil). Table 3 shows the results on FRAP and metal chelation activity of *O. americanum* and *O. basilicum* EOs. In ferric reducing and chelating metal ions, *O. basilicum* presented a higher FRAP (907.24 ± 18.29 mM Fe (II)/g oil) and metal-chelating (189.16 ± 09.04 mg EDTA Eq/g oil) activity.

**Table 3:** ABTS^•+^-scavenging, FRAP, and metal-chelating activities of EOs obtained from *O. basilicum* and *O. americanum* leaves

Sample	ABTS˙ + (μM TEAC/g oil)	FRAP (mM Fe (II) Eq/g oil)	Metal chelating activity (mg EDTA Eq/g oil)
*O. americanum*	2085.07 ± 7.43^c^	851.32 ± 13.71^c^	106.01 ± 12.07^c^
*O. basilicum*	2842.12 ± 10.39^b^	907.24 ± 18.29^b^	189.16 ± 09.04^b^
Ascorbic acid	4174.87 ± 12.65^a^	2365.65 ± 11.87^a^	312.65 ± 10.21^a^

Values are means of three independent analyses ± SD (*n* = 3). Mean values followed by different superscript letters (^a,b,c^) indicate significant statistical differences (*p* < 0.05)

### Antibacterial activity of *O. americanum* and *O. basilicum* EOs

**Fig. 3:**
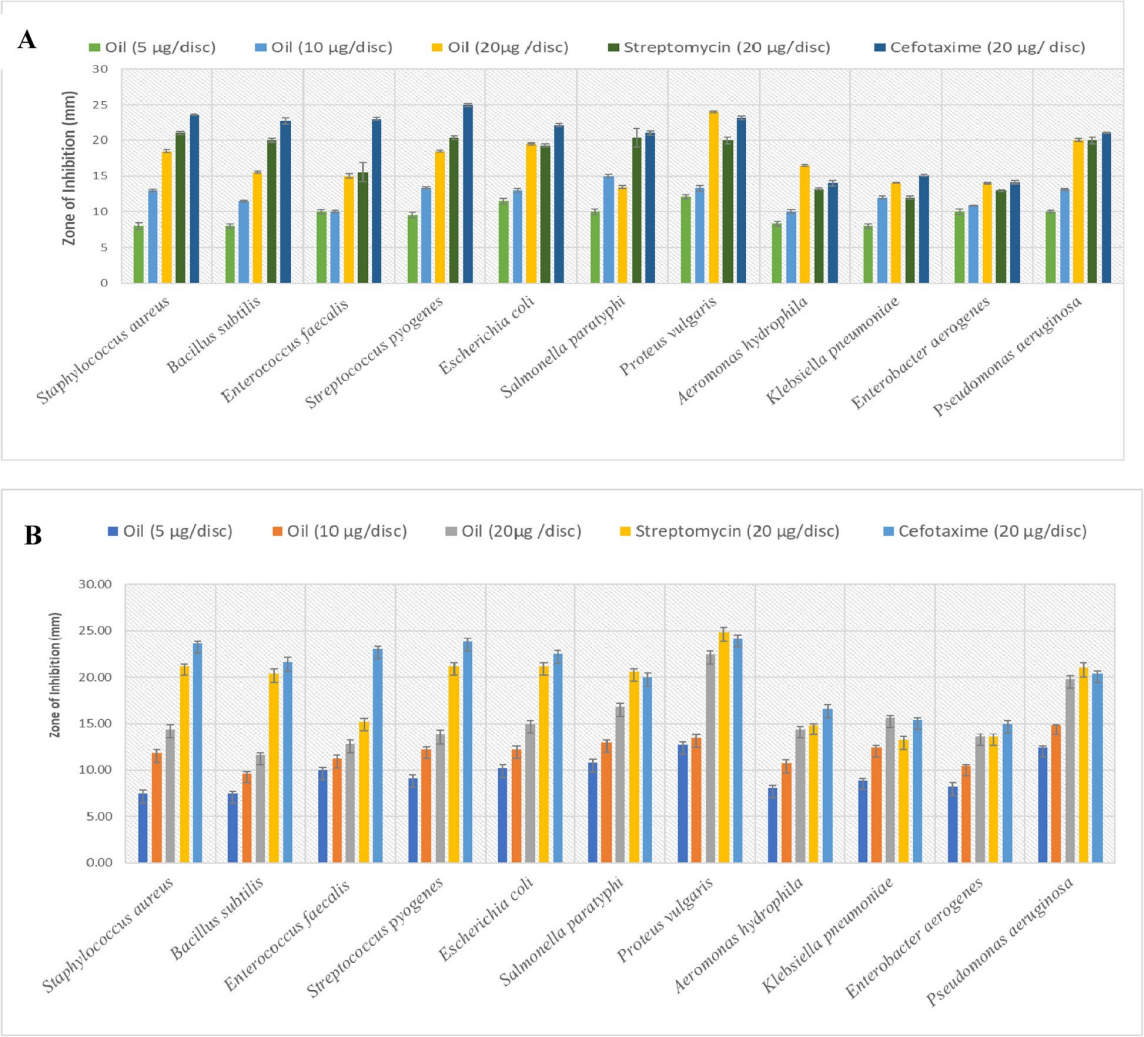
Antibacterial activity of EOs of *O. basilicum* and *O. americanum* leaves. **A** Antibacterial activity of *O. americanum* leaf EOs. **B** Antibacterial activity of *O. basilicum* leaf EOs. Values are means of three independent analyses ± SD (*n* = 3)

Figure 3 shows the ZI of *O. basilicum* and *O. americanum* oils evaluated via the disk-diffusion method.

The EOs obtained from *O. basilicum* and *O. americanum* displayed strong antibacterial activity against the tested pathogenic bacteria, with a ZI of 8.00–26.43 mm. Remarkably, the highest antibacterial activity was observed in *P. vulgaris within the* ZI of *O. basilicum* EO (26.43 mm) followed by that of *O. americanum* (24.00 mm), and such finding was comparable to that of streptomycin (20 µg/disk; 23.17 mm). Bactericidal activities against *S. paratyphi*, *E. aerogenes*, and *E. faecalis* ranged from 10.00 mm to 23.00 mm (Figs. 3A and 3B). In this case, the antibacterial activity of both plants exhibited dose dependence as it improved under a series of increased concentrations of the EO (5, 10, and 20 µg/disk).

The occurrence of live microorganisms in wells, which is an indication of colony formation, was confirmed by the decreases in yellow INT to triphenyl formazan (pink). This color was not detected in dead cells. The specific concentration of EO that can prevent the development of bacteria was investigated using this technique. The MIC and MBC of *O. basilicum* and *O. americanum* oils are displayed in Table 4 and differed from the range detected in the microdilution method (3.12 µg/mL to 100 µg/mL).

**Table 4:** MIC and MBC values of *O. americanum and O. basilicum* Eos

Microorganisms	*O. americanum*		*O. basilicum*		Cefotaxime	
	**MIC value (μg/mL)**	**MBC value ( μg/mL)**	**MIC value (μg/mL)**	**MBC value (μg/mL)**	**MIC value (μg/mL)**	**MBC value (μg/mL)**
*Staphylococcus aureus*	100.00	100.00	12.50	12.50	3.12	3.12
*Bacillus subtilis*	50.00	12.50	3.12	3.12	12.50	12.50
*Enterococcus faecalis*	100.00	100.00	12.50	12.50	4.88	3.12
*Streptococcus pyogenes*	50.00	50.00	6.25	6.25	3.12	2.40
*Escherichia coli*	9.76	12.50	3.12	3.12	3.12	3.12
*Salmonella paratyphi*	9.76	12.50	3.12	3.12	6.25	6.25
*Proteus vulgaris*	9.76	12.50	3.12	3.12	3.12	3.12
*Aeromonas hydrophila*	50.00	19.53	6.25	6.25	6.25	6.25
*Klebsiella pneumoniae*	50.00	50.00	6.25	6.25	3.12	3.12
*Enterobacter aerogenes*	100.00	100.00	12.50	12.50	6.25	6.25
*Pseudomonas aeruginosa*	50.00	12.50	6.25	6.25	12.50	12.50

Values are means of three independent analyses (*n* = 3)

### Larvicidal activity

The larvicidal efficiency of *O. basilicum* and *O. americanum* EOs was studied. The EOs had a notable larvicidal action, and the results are presented in Table 5.

**Table 5:** Larvicidal properties of leaf EOs from *O. americanum* and *O. basilicum* against the larvae of *A. aegypti* after 12 and 24 h of exposure

Sample	Mosquito life stages	Time	Concentration (ppm)	% mortality ± SD	LC_50_ (LCL-UCL)^a^	LC_90_ (LCL-UCL)^a^	χ2 (df = 4)^b^
*O. americanum*	Second instar	After 12 h	25	23.57 ± 0.42			
			50	33.88 ± 0.46	160.34	627.43	
			100	45.00 ± 1.00	(86.76–217.58)	(457.69–1202.18)	
			200	55.80 ± 0.83			5.406
			400	74.60 ± 1.14			
		After 24 h	25	23.56 ± 0.12			
			50	47.00 ± 0.69	87.96	439.54	10.45^*^
			100	64.30 ± 1.02	(12.70–163.11)	(292.16–716.18)	
			200	80.00 ± 1.50			
			400	91.54 ± 1.56			
	Third instar	After 12 h	25	11.80 ± 0.89			
			50	31.91 ± 1.53	134.54	1119.07	2.34
			100	45.00 ± 1.00	(46.72–227.60)	(781.28–2828.96)	
			200	57.80 ± 1.30			
			400	73.60 ± 0.89			
		After 24 h	25	21.20 ± 1.30			
			50	44.56 ± 1.28			
			100	58.20 ± 1.30	73.73(16.54–148.55)	577.28(353.49–961.49)	2.39^*^
			200	75.60 ± 0.54			
			400	82.60 ± 0.89			
*O. basilicum*	Second instar	After 12 h	25	32.20 ± 0.44			
			50	44.60 ± 1.14	61.14	1114.68	2.18
			100	53.20 ± 1.09	(17.76–342.99)	(242.16–3126.01)	
			200	67.20 ± 0.83			
			400	76.40 ± 1.14			
		After 24 h	25	41.60 ± 1.14			
			50	62.80 ± 1.92			
			100	71.40 ± 1.19	37.14	286.55	1.22*
			200	87.40 ± 1.14	(0.08–137.77)	(30.43–548.49)	
			400	93.20 ± 0.83			
	Third instar	After 12 h	25	27.40 ± 1.24			
			50	34.00 ± 0.87			
			100	56.76 ± 0.69	92.65	950.72	1.56
			200	69.10 ± 1.88	(0.03–259.13)	(495.34–3371.99)	
			400	78.00 ± 1.00			
		After 24 h	25	42.35 ± 1.20			
			50	64.50 ± 1.57			
			100	79.25 ± 1.65	31.43	233.43	1.09*
			200	86.00 ± 1.72	(0.41–111.35)	(37.22–459.36)	
			400	95.50 ± 1.89			

*LCL* Lower confidence level, *UCL* Upper confidence level

^a^95% confidence interval

^b^Degrees of freedom; χ2 chi-square value

^*^Significance at *P* ≤ 0.05

After 12 and 24 h of exposure, larvicidal effects, and mortality were observed in second- and third-instar larvae. As shown in Table 5, the larval mortality of *A. aegypti* (second and third instars) increased after the treatment using *O. basilicum* and *O. americanum* EOs at different concentrations (25–400 µg/mL). The treatment with *O. basilicum* at 25 g/mL caused 41.60% and 42.35% mortalities in the second- and third-instar larvae, respectively, and the values increased to 93.20% and 95.50% after a 24 h treatment period (Table 5).

After a 24 h exposure period, the LC_50_ of *O. basilicum* was the most effective against *Aedes aegypti* (LC_50_: 37.14 and 31.43 ppm and LC_90_: 286.55 and 233.43 ppm for the second and third instars, respectively, followed by that of *O. americanum* (LC_50_ 87.96 and 73.73 ppm LC_90_ 439.54 and 577.28 ppm). From the Chi-squared test, *O. basilicum* result showed significance chi-level χ2 = 1.09 and χ2 = 1.22 at *P* ≤ 0.05 (Table 5).

## Discussions

In *Ocimum* spp., EOs are generally rich in estragole and 2, 6-octadienal, as observed in our result for *O. basilicum* EO. The components vary from those reported by Mohamed Abdoul-Latif et al. [20], who reported linalool (41.2%) and estragole (30.1%) as the major compounds in *O. basilicum* from Djibouti in East Africa. The components also differed from those reported by Srivastava et al. [23], who observed methyl chavicol (51.2%–58.0%), methyl eugenol (10.0%–15.0%), meta-eugenol (4.5%–9.3%), and camphor (4.5%–5.7%) as the main chemotype II components in *O. basilicum* from India. The results of the EO analysis revealed methyl chavicol (88.6%) as the major compound of *O. basilicum* [42]. In contrast to our findings, some researchers reported that the main constituents of *O. basilicum* oil are 2,6-octadienal, 3,7-dimethyl-,(Z)-(20.01%), citral (20.11%), and eugenol (26.76) [43].

As shown in Table 2, the GC–MS analysis identified 34 compounds, which represent 100% of the total EOs in *O. americanum*. Interestingly, *O. americanum* oil was rich in camphor (33.86%) and limonene (7.21%). In contrast to our findings, Mohamed Abdoul-Latif et al. [20] reported that the main compounds in *O. americanum* EO include carvotanacetol (38.5%) and estragole (27.5%). Other investigators presented eugenol (45.2%), methyleugenol (14.8%), and (E)-caryophyllene (30.2%) as the major constituents of *O. americanum* from Rudrapur, India [44]. Similar to our result, camphor is a major chemical constituent of *O. minimum, O. canum,* and *O. gratissimum* but in different percentages [45–47]. These results suggest that the chemical composition of EOs in aromatic medicinal plants can vary depending on environmental factors and EO extraction methods [7, 48]. The different chemical profiles of the sources of plant materials affect the biological activity of EOs. Therefore, determining the chemical profiles of plant materials is essential for the quality control of their products.

EOs are capable natural scavengers that can diminish free-radical generation. They are offered as potential alternatives to artificial preservatives [48]. EOs react with the DPPH radical (deep-violet color) and alter it to hydrazine (DPPH-H discoloration). The extent of discoloration indicates the scavenging capability of samples [49]. This mechanism occurs due to the presence of antioxidants, which donate electrons/hydrogen atoms to stable radicals. Free-radical scavenging is generally indicated as the percentage of DPPH inhibition and the concentration essential for a 50% DPPH reduction (IC_50_). The present results indicated that *O. basilicum* oil had a more noticeable activity than *O. americanum* oil, with an IC_50_ value lower (11.56 ± 0.89 µg/mL) than that of *O. americanum* (13.42 ± 1.03 µg/mL). However, the standards, which included ascorbic acid (3.72 ± 0.60 µg/mL), rutin (4.65 ± 0.52 µg/mL), and BHT (7.89 ± 0.49 µg/mL), exhibited higher activities. These responses are consistent with those of Hazrati et al. [7] and Rezzoug et al. [27], who reported that EOs were less active than standard antioxidants.

Commonly, the antioxidant capability of oils is associated with their major bioactive constituents [50]. In a previous study, EOs, such as camphor, *α*-pinene, terpinene-4-ol borneol, eucalyptol,* p*-cymene, and* β*-pinene, were tested separately for their antioxidant activity, and the findings revealed their low free-radical scavenging capacities [51]. Zengin and Baysal [52] observed that linalool and α-terpineol have poor radical-scavenging capacity. Camphor, limonene, longifolene, caryophyllene, isoledene, *β*-citral, and estragole were the most abundant chemical constituents in *O. americanum* and *O. basilicum* in our study. This composition may clarify why oils display low antioxidant capability compared with synthetics. In numerous reports, the antioxidant capacity of EOs was determined by the occurrence of phenolic chemical constituents and synergistic effects of major chemical compounds, consistent with our results [7, 53–55]. EOs are a mixture of many chemical constituents derived from secondary metabolism. According to Tohidi et al. [56], oxygenated monoterpenes display high antioxidant, antifungal, and antibacterial activities. However, minor chemical constituents play an important role, and the constituents/combinations that are accountable for their antioxidant activities are not well known [57].

Usually, EOs and their chemical constituents are highly hydrophobic, which permits *bacteria* to lose their cell constituents and cause cell death [58]. The antibacterial activity of *O. basilicum* and *O. americanum* was studied in several bacteria. The agar-disk method is cheap, simple, and reproducible [59]. The EO of *O. basilicum* exhibited superior antibacterial activity. The MIC values of *O. basilicum oil* were lower than those of *O. americanum* oil. Similar observations were obtained for other EOs [60, 61]. In all tested organisms, the MBC was similar to the MIC values, which proved the antibacterial capability of these EOs. A similar observation was achieved in earlier research [59, 61].

The presence of oxygenated monoterpenes, such as geraniol formate, geraniol, citronellyl formate, citronellol, and linalool (64.93%–80.31%), as the main constituents is responsible for the EOs strong antibacterial activity against almost all susceptible microorganisms. According to specific studies, crude EOs have greater antibacterial activity than single compounds [62]. The antimicrobial activity of EOs has been linked to their hydrophobic nature, which allows them to penetrate the gram-positive bacterial cell membrane and inactivate molecular mechanisms that cause cell death [4, 7, 63].

Table 5 demonstrates that the larval mortality of *A. aegypti* (second and third instars) increased after the treatment with *O. basilicum* and *O. americanum* EOs at different concentrations (25–400 ppm). The treatment with *O. basilicum* EO at 25 ppm caused 41.60% and 42.35% mortalities in second- and third-instar larvae, respectively, and the values increased to 93.20% and 95.50% after a 24 h treatment period (Table 5). A similar tendency has been observed for both instars of *A. aegypti* at different concentrations of *O. americanum* EOs at 12 and 24 h. Mortality was dose-dependent in our study. Earlier reports showed agreement with our statement [64–66]. According to our findings, the chi-square value in *O. basilicum* for second-instar and third-instar larvicidal activity was significant at *p* ≤ 0. 05. This result indicates that *O. basilicum* essential oil was potent against *A. aegypti* (dengue fever vector). Likewise, Eos from *Artemisia vulgaris* showed the chi-square value (χ^2^ = 3.14) for third-stage *A. aegypti* [67]. The results for larvicidal capability revealed that the ratio of deaths was directly proportional to the oil concentration which demonstrated that concentration plays a vital role in larvicidal activity. In all the analyses, the mortality was found to increase when the concentration increased. The higher the concentration of toxic substances, the greater the potential of their toxic effect was observed. EOs isolated from plants have been documented as botanical pesticides [68]. The EO of *Ipomoea cairica* exhibited 100% mortality against *Anopheles subpictus, Culex tritaeniorhynchus*, and *Aedes albopictus* [69]. Tiwary et al. [70] perceived the larvicidal effects of linalool against *A. stephensi* (LC_50_ = 58 ppm), *C. quinquefasciatus* (LC_50_ = 49 ppm), and *A. aegypti* (LC_50_ = 54 ppm). Cheng et al. [71] observed that 36.0–86.8 µg/mL EOs were required to kill *A. aegypti*. Cavalcanti et al. [72] determined that the larvicidal efficacy of Brazil oils against *Aedes aegypti* ranged from 60 µg/mL to 69 µg/mL. Rahuman et al. [73] also perceived that *Feronia limonia* dried leaves were associated with high activity against *C. quinquefasciatus*, *A. stephensi,* and *A. aegypti*. The larvicidal efficacy of *Thymus vulgaris, Mentha arvensis, Cymbopogan citratus, P. graveolens,* and *O. basilicum* EOs were investigated against *C. quinquefasciatus*. Cheng et al. [71] reported that 1,8-cineole and eugenol from *O. gratissimum* had LC_50_ of 60 ppm against *A. aegypti* larvae. A similar activity was observed on the oils of *O. basilicum* and *O. americanum.* Early researchers identified the efficiency of EOs against mosquito larvae and adults [74]. Moreover, previous studies noted the larvicidal activity of EOs isolated from different plants, such as the Rutaceae plants [75] *Eucalyptus globulus* [76] and *Syzygium* [77]. Other works reported that the EOs extracted from different plants show characteristic chemical composition and affect various biological properties [77–81]. For example, the four main constituents of Rutaceae (*Citrus aurantiumare*) oil, namely, diethyl o-phthalate, limonene, methyl dihydrojasmonatem, and limonene, exhibit the highest larvicidal activity against *Aedes albopictus* [75]. The five major components identified in *Eucalyptus globulus* oil are 1,8-cineole (or eucalyptol), α-pinene, *trans*-pinocarveol, aromadendrene, and globulol. These compounds showed a potential larvicidal activity against *Anopheles stephensi* [76]. Compared with *O. americanum* and *O. basilicum* oils, some major chemical components, such as camphor, estragole, levomenol, and veridiflorol, differ from those in other works [75–77]. In this study, several factors were responsible for the good larvicidal activity against *Aedes aegypti.* Mainly, the activity may be related to their major chemical components in both oils, and the minor components can also contribute by acting in a synergetic manner [77]. In addition, the age and distribution of plants can affect larvicidal activity [81].

## Conclusions

The results demonstrated that camphor, limonene, longifolene, caryophyllene and estragole are the most abundant components in *O. basilicum* and *O. americanum.* This study discovered that the oils of both plants have strong antibacterial and larvicidal activities but a low antioxidant activity. These EOs should be further evaluated to develop safe agents for larvicidal therapy. Further studies should be conducted to reveal the mode of action of these oils to understand their possible roles in human wealth.

## Data Availability

All relevant data are included within the manuscript and are available from the corresponding author upon reasonable request.

## Abbreviations

EO: Essential oil
GC–MS: Gas chromatography–mass spectrometry
*O. basilicum*: *Ocimum basilicum*
O. americanum: *Ocimum americanum*
DPPH: 2,2-Diphenylpicrylhydrazyl
FRAP: Ferric reducing antioxidant power
ABTS: 2,2′-Azino-bis (3-ethylbenzothiazoline-6-sulfonic acid
MIC: Minimal inhibitory concentration
MBC: Minimum bactericidal concentration
LC_50_ and LC_90_: 50% And 90% lethal concentration, respectively
IC_50_: Half-maximal inhibitory concentration
ppm: Parts per million
BHT: Butylated hydroxytoluene
ZI: Zone of inhibition
EDTA: Ethylenediaminetetraacetic acid
